# Gene Mutation Characteristics and Prognostic Significance in Acute Myeloid Leukemia Patients From Northeast China

**DOI:** 10.1155/humu/7730186

**Published:** 2025-02-20

**Authors:** Yiyang Shen, Shuang Fu, Xuan Liu, Jianing Liu, Yu Fu, Yue Zhao, Xinxin Wang, Xujian Jiang, Jihong Zhang

**Affiliations:** Hematology Laboratory, Shengjing Hospital of China Medical University, Shenyang, China

**Keywords:** acute myeloid leukemia, gene mutations, next-generation sequencing

## Abstract

A great part of studies on the correlation between gene mutations and prognosis in acute myeloid leukemia (AML) patients are based on Western populations. To profile the genomic landscape of AML patients in Northeast China, we retrospectively analyzed the clinical data of 377 newly diagnosed AML patients in Shengjing Hospital of China Medical University from 2016 to 2022 and compared them with data from other populations with different genetic backgrounds. The mutation status of *NPM1*, *FLT3-ITD*, *FLT3-TKD*, *CEBPA* (CCAT enhancer binding protein alpha), *ASXL1*, *TET2*, *KIT*, *DNMT3A* (DNA methyltransferase 3A), *IDH1*, *IDH2*, *EZH2* (enhancer of zeste 2), *RUNX1*, *TP53*, *NRAS*, and *GATA2* was acquired by next-generation sequencing (NGS) technology; meanwhile, the clinical data of the patients were collected. The Cox regression model was used to analyze factors affecting patient survival and the impact of *CEBPA* and *DNMT3A* mutation on prognosis, and the results were different from those in other populations. Seventy-seven of 377 patients (20.4%) were detected with *CEBPA* mutations, which was higher than the 2%–6% in the Caucasian population. In the *CEBPA^dm^* patients who did not receive bone marrow transplantation, the prognosis of male patients (*n* = 18) was significantly better than that of female patients (*n* = 21) (*p* = 0.0242). Sixty-three of 377 patients (16.7%) carried the *DNMT3A* mutation, which was lower than the mutation frequency of 20.9% in the German–Austrian population, and the prognosis of these patients was significantly poorer (*p* = 0.0052). In addition, the prognostic evaluation value of the *DNMT3A* mutation in AML patients was not affected regardless of the presence of the *NPM1* and *FLT3-ITD* comutation (*p* > 0.05), nor the mutation site of *DNMT3A*. In conclusion, for the Northeastern Chinese population, the prognosis of male patients with *CEBPA^dm^* was more favorable than that of female patients, and the *DNMT3A* mutation serves as an independent predictor of poor prognosis in AML. These results highlighted the central role of genetic background in precision medicine strategies and further emphasized the importance of the clinical characteristics of AML gene mutations in the Chinese population.

## 1. Introduction

Acute myeloid leukemia (AML) is a malignant disease characterized by abnormal clonal proliferation and differentiation arrest of hematopoietic stem cells or progenitor cells [1]. The latest Chinese cancer data shows that the mortality rate of leukemia in China's population is 4.03/100,000 [2]. Next-generation sequencing (NGS)–based analysis has elucidated key gene mutations commonly found in AML patients [3]. According to the 2022 European Leukemia Network (ELN) guideline, *NPM1* without *FLT3-ITD* mutation and in-frame *CEBPA* (CCAT enhancer binding protein alpha)–basic leucine zipper (*CEBPA^bZip-inf^*) mutation are considered favorable prognostic indicators, while *TP53* mutation represents an unfavorable prognostic factor. *ASXL1*, *BCOR*, enhancer of zeste 2 (*EZH2*), *RUNX1*, *SF3B1*, *SRSF2*, *STAG2*, *U2AF1*, and/or *ZRSR2* mutations are considered adverse prognostic markers when not coexisting with favorable-risk AML subtypes [4]. Ethnic backgrounds may affect the molecular driving factors of disease development and progression, whether in solid tumors or leukemias [5–7]. For most of the current studies, which are based on data from European and American populations, the established prognostic classification criteria are based on their genetic characteristics. For the Chinese population, especially those in the Northeast, there is currently a lack of a comprehensive genomic landscape to establish prognostic classification criteria.

Some gene mutations have been well-studied, leading to the development of corresponding targeted drugs, such as inhibitors for Fms-like tyrosine kinase 3 (*FLT3*), *EZH2*, and isocitrate dehydrogenases 1 and 2 (*IDH1/2*) [8–11]. However, it is worth noting that though some mutations are frequently observed in AML, they have not demonstrated consistent frequency and prognostic significance in studies conducted in different countries [12–14]. Therefore, we included 15 gene mutations in our analysis, with a particular focus on *CEBPA* and *DNMT3A* (DNA methyltransferase 3A). Our aim is to comprehensively characterize the genetic mutation profiles of AML patients in Northeast China, providing more detailed information for clinical practice in Chinese AML patients' treatment.

The coding gene of *CEBPA* is located on Chromosome 19. Its protein contains two transactivation domains (TADs) at the N-terminal and a basic leucine zipper region (bZIP) at the C-terminal [15, 16]. In previous studies, biallelic mutations in the *CEBPA* gene have been defined as the marker of favorable prognosis. However, Taube et al. found that patients with *CEBPA^bZIP-inf^* had better complete remission rates and longer overall survival (OS) and event-free survival (EFS) than patients with other *CEBPA* mutations [17]. Study results on children and young adults also support this conclusion [18].

The *DNMT3A* gene is located on Chromosome 2p23 [19]. *DNMT3A* mutations are found in about 20% of AML patients [20]. Patients with *DNMT3A* mutations appeared to be older, often with normal karyotypes, and more common in M4 and M5 [21]. R882 is the most common site of *DNMT3A* mutations [22]. The prognostic impact of *DNMT3A* mutations, though commonly in AML, is not fully revealed, and the ELN 2022 classification did not include it as a prognostic indicator [4]. In studies conducted in different regions, the results of *DNMT3A* on prognosis are not consistent [20, 23–25]. Therefore, even for well-studied gene mutations, the impact of ethnic background on prognosis is not neglectable.

In this study, we collected the gene mutations and clinical information of 713 AML patients in Northeast China and further analyzed the impact of these gene mutations on survival in 377 cases with follow-up information. We focused on the differences in *CEBPA* and *DNMT3A* mutations between AML patients in the Northeast Chinese and Western populations and demonstrated that genetic background differences are important factors affecting the prognostic stratification of AML patients. Additionally, we also compared the impact of the ELN 2017 and 2022 versions on patient stratification. The results indicated that the 2022 version exhibited superior efficacy. This article provides a reference for the diagnosis and treatment of AML in the Northeast Chinese population.

## 2. Patients, Materials, and Methods

### 2.1. Patient Samples

All bone marrow (BM) samples and peripheral blood (PB) samples were obtained from 713 patients diagnosed with AML from 2016 to 2022 in the Hematology Laboratory of Shengjing Hospital Affiliated to China Medical University. Of 377 patients with available follow-up information after treatments, 126 patients were alive and 252 were deceased before 8-19-2023. The study was approved by the ethics board of the Shengjing Hospital of China Medical University.

### 2.2. Treatment

Most patients were treated with an IA (idarubicin and cytarabine), and partial patients were treated with a DA (daunorubicin and cytarabine). Considering the age factor, a small number of elderly patients were treated with CAG (cytarabine, aclarubicin, and granulocyte colony-stimulating factor), DCAG (decitabine, cytarabine, aclarubicin, and granulocyte colony-stimulating factor), and other regimens.

### 2.3. NGS

Total RNA was extracted from BM cells of AML patients at the time of initial diagnosis using the TRIzol method, and a cDNA library was prepared. Genomic DNA was extracted from the BM samples using the Genomic DNA Extraction KIT (Tiangen, Beijing, China). In general, 200 ng of genomic DNA was used to create the sequencing library using the MultipSeq Custom Panel (Rightongene, Shanghai, China). Targeted NGS was performed on the Novaseq platform (Illumina, California, United States) with a sequencing depth of 1000×.

### 2.4. Statistical Analysis

The relationship between categorical variables was analyzed using *χ*^2^ or Fisher's exact test. Independent sample *t*-test or Mann–Whitney *U* test was used to compare the differences between groups of continuous variables. All tests were two-sided, and *p* < 0.05 was considered statistically significant. OS was calculated from the date of diagnosis to death, and the OS of patients who were still alive before the end of the follow-up date was from diagnosis to the follow-up deadline. The Cox proportional hazards model was used for survival analysis. The log-rank test was used to determine the difference in survival distribution, and the significant threshold was *p* < 0.05. All statistical analyses were performed using GraphPad Prism 9.0 software and the SPSS software package, Version 26 (SPSS, Chicago, Illinois).

## 3. Results

### 3.1. Characteristics of Clinical Data in AML Patients

We conducted a comprehensive analysis of 713 newly diagnosed AML patients in our hospital. As shown in Table 1, the median age was 55 (range 42–64), the median of BM blast cells was 58.3% (range 38.4%–75.6%), the median of white blood cells (WBC) was 14.6 × 10^9^/L (range 3.8‐48.8 × 10^9^/L), the median of platelets (PLT) was 40 × 10^9^/L (range 18‐79.75 × 10^9^/L), and the median of hemoglobin (HGB) was 77 g/L (range 63.25–97 g/L). There was no difference between males and females in these indicators (*p* > 0.05), except that the HGB level was higher in males than in females (80 g/L vs. 74.5 g/L, *p* = 0.0004). The most common AML subtype was M2 (38.8%), followed by M5 (32.3%), M4 (21.5%), M1 (6.0%), unclassified AML (0.6%), M0 (0.4%), M6 (0.3%), and M7 (0.1%). *NPM1* (21.7%), *FLT3-ITD* (20.3%), *CEBPA* (18.4%), *NRAS* (17.0%), *TET2* (16.4%), and *DNMT3A* (16.4%) were the most common mutations in AML. The proportion of males with NRAS mutations was higher than that of females (Table 1, 19.9% vs. 14.0%, *p* = 0.022).

**Figure 1:**
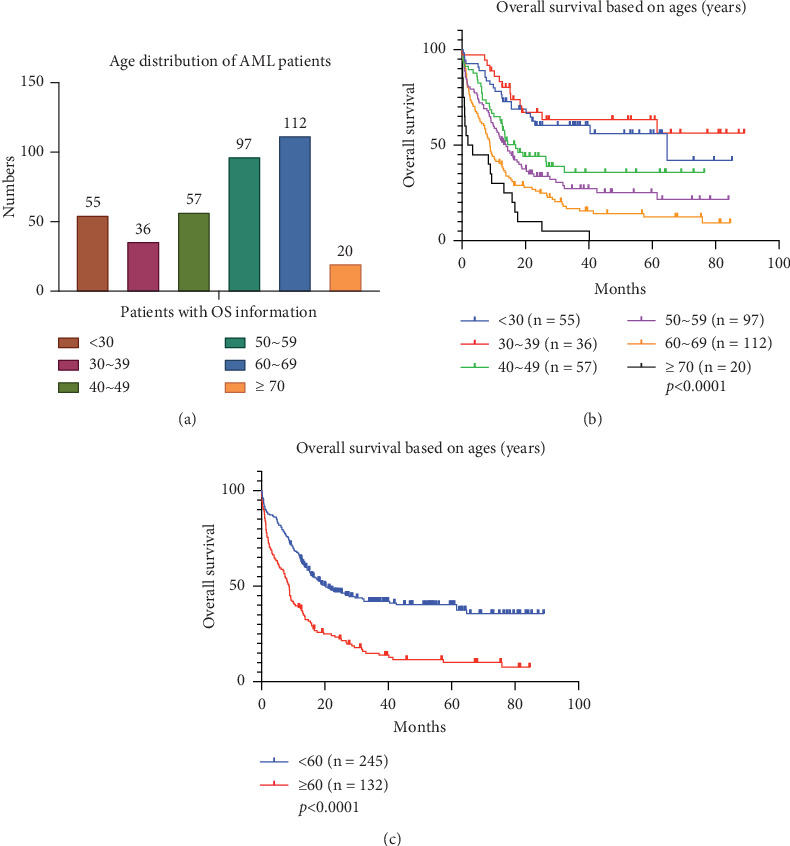
Analysis of age distribution and overall survival (OS) in Northeast Chinese AML patients. This analysis includes a total of 377 cases with comprehensive follow-up information, with patients grouped by decade of age for comparison. (a) Age distribution of 377 patients. (b) OS of Northeast Chinese AML patients diagnosed and treated at Shengjing Hospital. (c) Comparison of OS between patients aged below 60 years and those aged 60 years and above.

Of all 713 patients, 377 had available follow-up information, and subsequent analysis of factors affecting survival was based on the clinical information of this part of patients. To gain a deeper insight into the age characteristics of AML in this region, we referred to the literature and categorized 377 patients aged between 14 and 86 years into age intervals of 10 years each [17]. Among which, 60–69 is the group with the highest proportion, counting for 29.7%. Among other age groups, 14.6% of the patients were under 30 years old, 9.5% were between 30 and 39 years old, 15.1% were between 40 and 49 years old, and 25.7% were between 50 and 59 years old. Only 5.3% of the patients were over 70 years old (Figure 1(a)). In the analysis of OS stratified by age, a prognosis was observed of a worsening trend along with age (Figure 1(b), *p* < 0.001). However, there was no significant difference in prognosis between patients under 30 years old and 30–39 years old (*p* = 0.4847) (Figure S1). According to the guidelines of the Chinese Society of Clinical Oncology, 60 years old is the cutoff point for treatment regimen changes. Our study observed that the OS of patients aged ≥ 60 years was significantly lower than that of patients < 60 years old (Figure 1(c), *p* < 0.001), which was consistent with the Chinese guideline.

### 3.2. Male Patients With *CEBPA^dm^* Mutations Have a Better Prognosis Than Those of Female Patients

**Figure 2:**
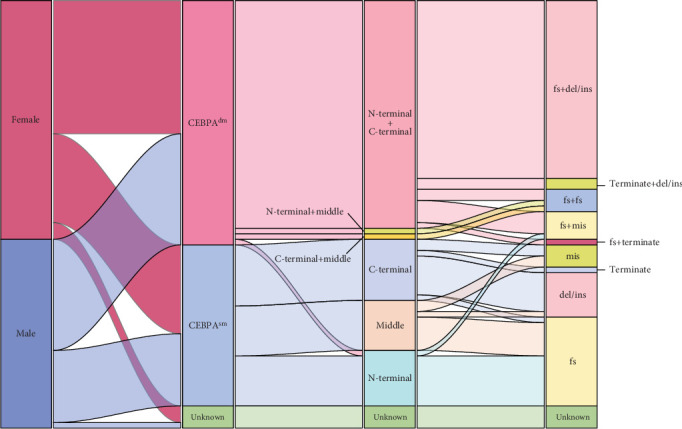
Sankey diagram illustrating the interrelationships among sex, *CEBPA* status, mutation sites, and mutation types in 77 AML patients. The diagram, read from left to right, visually maps the complex interconnections between patient characteristics and genetic alterations. Each distinct rectangle represents a unique patient subgroup, with the size of the rectangle directly proportional to the patient counts within those groups. The thickness of the connecting lines reflects the frequency of transitions between different categories. Fs, frameshift; del, deletion; ins, insert; mis, missense.

**Figure 3:**
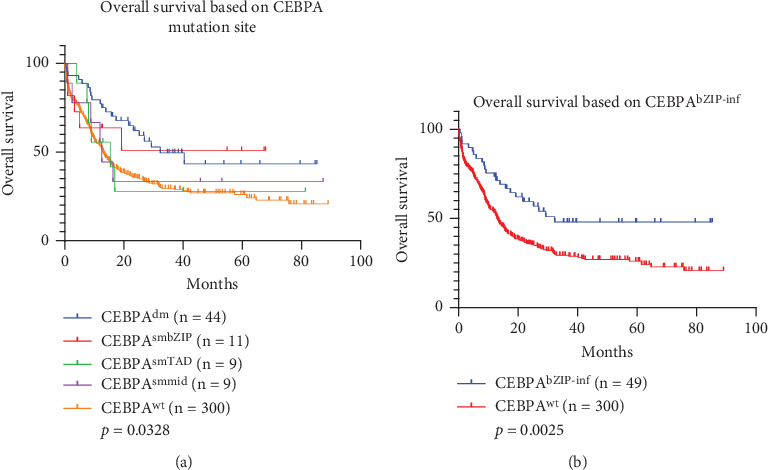
Survival analysis based on *CEBPA* mutation status within Northeast Chinese AML patients. (a) OS of AML patients with different *CEBPA* mutation status (*CEBPA^dm^*, *CEBPA^smbZIP^*, *CEBPA^smTAD^*, *CEBPA^smmid^*, and *CEBPA^wt^*). (b) Comparison of OS between patients with *CEBPA^wt^* and those with in-frame mutations within *CEBPA^bZIP-inf^*.

According to gene structure, the *CEBPA* mutation was distributed in three regions: N-terminal (aa 1-120), middle (aa 121-277), and C-terminal (aa 278-358) [16]. As shown in Figure 2, in the 377 patients with follow-up information, *CEBPA* mutations were observed in 20.42% of patients (77/377). Further analysis showed that 11.67% (44/377) were *CEBPA^dm^*, and the vast majority were double mutations in the N-terminal and C-terminal regions. Meanwhile, a double mutation in the N-terminal and the middle regions, a double mutation in the C-terminal and the middle regions, and a double mutation in the N-terminal were also detected. 7.69% (29/377) of these mutations belonged to the *CEBPA^sm^*. Among them, 11 were *CEBPA^smbZIP^*, nine were *CEBPA^smTAD^*, and nine were *CEBPA^smmid^*. In the past, our research adhered to the ELN 2017 guideline [26]. However, with the release of the ELN 2022 guideline, which designates *CEBPA^bZIP-inf^* as a marker of good prognosis, we have revisited and reassessed our research in light of this new evidence [4]. *CEBPA^bZIP-inf^* covers patients with C-terminal mutations in *CEBPA^dm^* (*n* = 39), as well as the *CEBPA^smbZIP^* patients (*n* = 10). The survival analysis plots of different *CEBPA* mutation types showed that *CEBPA^dm^* and *CEBPA^smZIP^* were with a relatively better prognosis (Figure 3(a), *p* = 0.0328). Regardless of whether other *CEBPA* mutations are combined, *CEBPA^bzip-inf^* mutations were good prognostic indicators in the Northeast Chinese population (Figure 3(b), *p* = 0.0025, hazard ratio (HR): 0.5913, 95% confidence interval (CI): 0.4205–0.8315).

**Figure 4:**
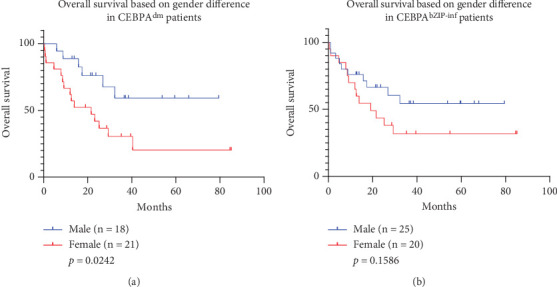
Survival analysis according to the *CEBPA* mutation subtypes who did not undergo allogeneic hematopoietic stem cell transplantation. (a) Comparison of OS rates between male and female *CEBPA^dm^* patients. (b) Comparison of OS rates between male and female *CEBPA^bZIP-inf^* patients.

In order to analyze the impact of gender on prognosis, we grouped *CEBPA* mutations by gender. As shown in Figure 4, after excluding the patients who received allogeneic transplantation, we found that in the *CEBPA^dm^* mutations, males had a significantly better prognosis (median OS 25.3 months, range 15.33–52.24 months) than females (median OS 19.0 months, range 8.25–32.3 months) (Figure 4(a)*p* = 0.0242, HR: 0.3724, 95% CI: 0.1577–0.8792). All clinical examination data differences between males and females are detailed in Table 2. None of these patients had fusion gene expression. There were no significant differences in other clinical examinations between males and females (*p* > 0.05), but among the *CEBPA^dm^* patients, 19.0% of female patients had a slightly higher proportion of chromosomal changes compared to 5.6% of male patients. In 18 male patients, 16 patients had normal karyotypes, one patient did not perform karyotype analysis, and one patient showed an abnormal karyotype of 46,XY,del(3)(p22),del(7)(p14),-9,add(10)(q25),+mar. In 21 female patients, four had abnormal karyotypes with 46,XX,add(7)(q21), 47,XX,+8, 46,XX,i(7)(q10), and 47,XX,+21, respectively. Two cases did not perform karyotype analysis. Chromosomal abnormalities alone may not fully explain the poorer prognosis observed in female patients. Our data showed that of the 21 female patients, 15 unfortunately died, and eight of them survived for less than a year. Therefore, it is necessary to include more cases for further research and analysis to fully understand the potential factors leading to poorer prognosis in female patients in the future.

Recent studies have shown that the *CEBPA^bZIP-inf^* mutation is a good prognostic indicator, similar to *CEBPA^dm^*. However, when we performed Cox analysis by grouping the *CEBPA^smbZIP-inf^* patients by gender, male and female patients did not show significant differences (Figure 4(b), *p* = 0.1586, HR: 0.5503, 95% CI: 0.2399–1.262), which is different from the impact of gender on prognosis in *CEBPA^dm^* patients. Previously described male patients with *CEBPA^dm^* tend to have normal karyotypes. The patients in the *CEBPA^bZIPsm-inf^* group mostly overlapped with the *CEBPA^dm^* group, though the *CEBPA^bZIPsm-inf^* group was with an additional patient harboring an abnormal karyotype of 45,XY,-9.

**Figure 5:**
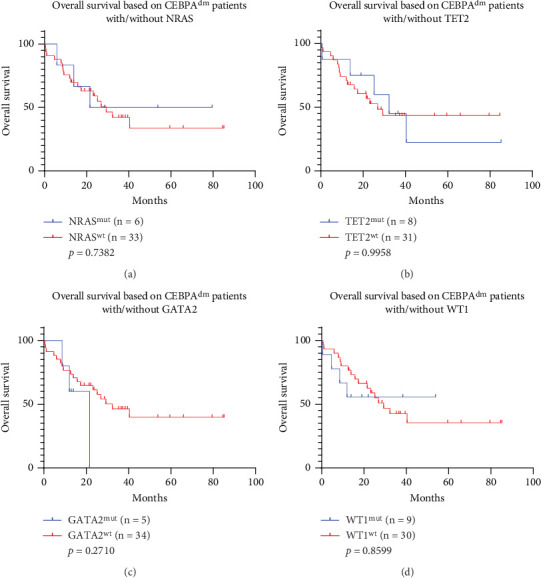
Outcome analysis of *CEBPA^dm^* patients with *NRAS*, *TET2*, *GATA2*, or *WT1* mutations. (a) Comparison of OS rates between *CEBPA^dm^* patients with *NRAS^mut^* and without *NRAS* mutations (*NRAS^wt^*). (b) Comparison of OS rates between *CEBPA^dm^* patients with *TET2^mut^* and without *TET2* mutations (*TET2^wt^*). (c) Comparison of OS rates between *CEBPA^dm^* patients with *GATA2^mut^* and without *GATA2* mutations (*GATA2^wt^*). (d) Comparison of OS rates between *CEBPA^dm^* patients with *WT1^mut^* and without *WT1* mutations (*WT1^wt^*).

To analyze the correlation between *CEBPA* mutational status and other molecular mutations, we also analyzed several gene mutations that frequently co-occur with *CEBPA* mutant patients, including *NRAS*, *TET2*, *GATA2*, and *WT1.* Comutations of *CEBPA* and these genes did not affect the prognosis (Figures 5(a), 5(b), 5(c), and 5(d), *p* > 0.05).

### 3.3. *DNMT3A* Can Independently Predict Poor Prognosis

**Figure 6:**
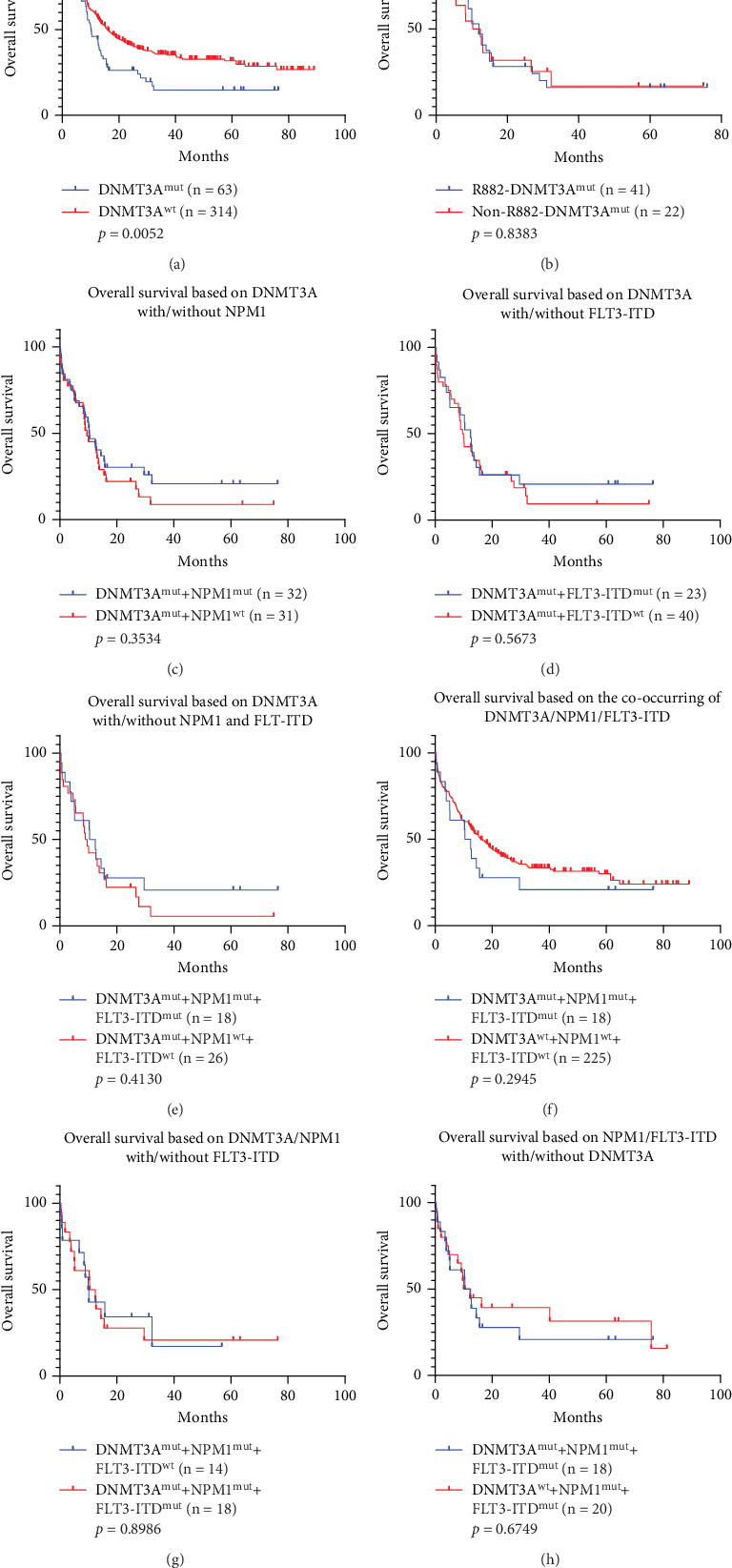
Outcome analysis of *DNMT3A* mutant patients with *NPM1* or *FLT3-ITD* mutations. (a) OS rates of the *DNMT3A^mut^* patients compared to those with *DNMT3A^wt^* patients. (b) OS comparison between the patients with *DNMT3A* R882 mutations and without *DNMT3A* R882 mutations in the cohort of *DNMT3A* mutation patients. (c) OS comparison between patients with dual *DNMT3A^mut^*+*NPM1^mut^* and those with *DNMT3A^mut^*+*NPM1^wt^*. (d) OS comparison between patients with dual *DNMT3A^mut^*+*FLT3-ITD^mut^* and those with *DNMT3A^mut^*+*FLT3-ITD^wt^.* (e) OS comparison between patients with *DNMT3A^mut^*+*NPM1^mut^*+*FLT3-ITD^mut^* and those with *DNMT3A^mut^*+*NPM1^wt^*+*FLT3-ITD^wt^*. (f) OS comparison between patients with *DNMT3A^mut^*+*NPM1^mut^*+*FLT3-ITD^mut^* and those with *DNMT3A^wt^*+*NPM1^wt^*+*FLT3-ITD^wt^*. (g) OS comparison between patients with *DNMT3A^mut^*+*NPM1^mut^*+*FLT3-ITD^wt^* and those with *DNMT3A^mut^*+*NPM1^mut^*+*FLT3-ITD^mut^*. (h) OS comparison between patients with *DNMT3A^mut^*+*NPM1^mut^*+*FLT3-ITD^mut^* and those with *DNMT3A^wt^*+*NPM1^mut^*+*FLT3-ITD^mut^.*

In all 713 patients, 117 (16.4%) had *DNMT3A* mutations. In the 377 patients with follow-up information, 63 (16.7%) had *DNMT3A* mutations, which were associated with a worse prognosis (Figure 6(a), *p* = 0.0052, HR: 0.6375, 95% CI: 0.4688–0.867). The impact of the *DNMT3A* mutation on patient prognosis was not related to the R882 site (Figure 6(b), *p* = 0.8383). Compared with wild-type patients, *DNMT3A*-MUT patients were older (Table 3, 59 years [53, 63] vs. 53 years [36, 62], *p* < 0.001) and had higher WBC (Table 3, 33.9 ×10^9^/L [9, 64.6] vs. 11.25 ×10^9^/L [3.3, 43.87], *p* = 0.0015) and PLT (Table 3, 58 ×10^9^/L [24, 97] vs. 38 ×10^9^/L [17, 70], *p* = 0.0048). *DNMT3A* mutations occurred more frequently in M5 subtype patients and were more often combined with *FLT3-ITD* or *NPM1* mutation. However, coexisting with *NPM1* or *FLT3-ITD* mutation did not affect the OS of patients solely with *DNMT3A* mutation (Figures 6(c), 6(d), 6(e), 6(f), 6(g), and 6(h), *p* > 0.05).

### 3.4. *TP53* and *ASXL1* Can Well Predict Poor Outcomes

**Figure 7:**
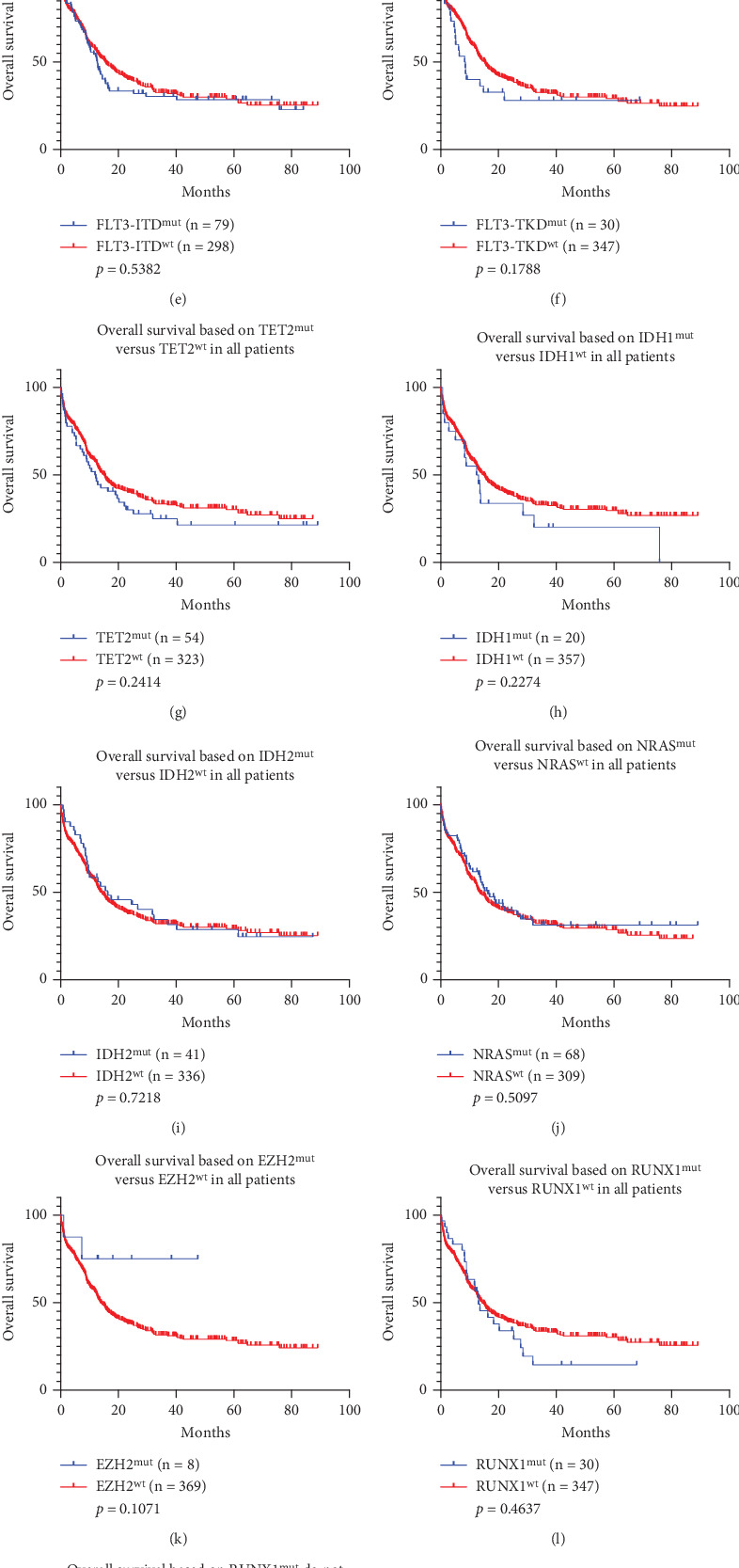
Survival analysis according to different gene mutations in 377 patients. (a) OS of *NPM1^mut^* versus *NPM1^wt^* patients. (b) OS of *NPM1^mut^* versus *NPM1^wt^* in patients with a normal karyotype. (c) OS analysis of different combinations of *NPM1* and *FLT3-ITD* mutations in patients with a normal karyotype, including *NPM1^mut^*+*FLT3-IT^mut^*, *NPM1^mut^*+*FLT3-ITD^wt^*, and *NPM1^wt^*+*FLT3-ITD^wt^.* (d) OS of *NPM1^mut^*+*NRAS^mut^* versus *NPM1^wt^*+*NRAS^wt^* patients. (e) OS of *FLT3-ITD^mut^* versus *FLT3-ITD^wt^* patients. (f) OS of *FLT3-TKD^mut^* versus *FLT3-TKD^wt^* patients. (g) OS of *TET2^mut^* versus *TET2^wt^* patients. (h) OS of *IDH1^mut^* versus *IDH1^wt^* patients. (i) OS of *IDH2^mut^* versus IDH2^wt^ patients. (j) OS of *NRAS^mut^* versus *NRAS^wt^* patients. (k) OS of *EZH2^mut^* versus *EZH2^wt^* patients. (l) OS of *RUNX1^mut^* versus *RUNX1^wt^* patients. (m) OS of *RUNX1^mut^* do not co-occur with favorable-risk AML subtypes versus *RUNX1*^wt^. (n) OS of *KIT^mut^* versus *KIT^wt^* in patients. (o) OS of *ASXL1^mut^* versus *ASXL1^wt^* patients. (p) OS of *TP53^mut^* versus *TP53^wt^* patients.

We also performed univariate Mantel–Cox regression analysis on the correlation between the other gene mutations and OS and found that *NPM1*, *FLT3-ITD*, *FLT-TKD*, *TET2*, *IDH1*, *IDH2*, *NRAS*, *EZH2*, *RUNX1*, or *KIT* mutations did not affect prognosis (*p* > 0.05, Figures 7(a), 7(b), 7(c), 7(d), 7(e), 7(f), 7(g), 7(h), 7(i), 7(j), 7(k), 7(l), 7(m), and 7(n)). But *ASXL1* (Figure 7(o), *p* = 0.0072, HR: 0.6755, 95% CI: 0.4726–0.9654) or *TP53* (Figure 7(p), *p* < 0.001, HR: 0.08497, 95% CI: 0.1457) mutations were associated with poorer prognosis.

Multivariable analysis using the Cox proportional hazards model was conducted to estimate the impact of factors such as patient age, gender, WBC, PLT, HGB, BM blast, and PB blast on patient survival rates. Initially, univariate regression analyses were performed for each variable individually, and variables with a *p* < 0.15 in the univariate analysis were selected for inclusion in the multivariate analysis. The results of the univariate Cox analysis are presented in Table 4.

Based on the results of the Cox univariate regression analysis of clinical data presented in Table 4 and Figure 7, we have included variables with *p* values less than 0.15 in the subsequent multivariate analysis. It is noteworthy that, although in our analysis, *NPM1* (Figure 7(a), *p* = 0.5401, HR: 0.9125, 95% CI: 0.6808–1.223) and *FLT3-ITD* (Figure 7(e), *p* = 0.5382, HR: 1.101, 95% CI: 0.8105–1.495) did not reach statistical significance, we have decided to include these two factors in the multivariate Cox analysis as well. This decision is based on their well-established prognostic impact on patients in clinical practice and previous studies. The results of the multivariate Cox analysis are shown in Table 5.

We found that HGB levels (Table 5, *p* = 0.003, HR: 0.991, 95% CI: 0.986–0.997), *NPM1* mutation (Table 5, *p* = 0.006, HR: 0.629, 95% CI: 0.452–0.877), and *CEBPA^bZIP-inf^* mutation (Table 5, *p* = 0.007, HR: 0.533, 95% CI: 0.337–0.841) were independent favorable prognostic factors for AML patients. In contrast, age (Table 5, *p* < 0.001, HR: 1.037, 95% CI: 1.027–1.047), percentage of BM blasts (Table 5, *p* = 0.023, HR: 1.009, 95% CI: 1.001–1.016), and *TP53* mutations (Table 5, *p* < 0.001, HR: 3.701, 95% CI: 2.092–6.547) were identified as independent unfavorable prognostic factors for AML patients. Although the results of *DNMT3A* mutations did not show statistical significance (Table 5, *p* = 0.059), it still indicated an adverse effect on the survival of AML patients (Table 5, HR: 1.371, 95% CI: 0.987–1.904). Meanwhile, *FLT3-ITD* and *ASXL1* mutations did not demonstrate an adverse effect on prognosis in our data.

#### 3.5. Survival Outcomes According to ELN 2017 and 2022 Guidelines

Based on the ELN 2017 and 2022 guidelines, we conducted a detailed and accurate risk stratification for patient prognosis and compared the stratification results of the two guideline versions. After excluding cases with incomplete clinical and follow-up information, a total of 332 samples were included in the ELN 2017 prognosis stratification system. Among them, 142 (42.8%) patients were classified as favorable-risk, 98 (29.5%) patients were classified as intermediate-risk, and 92 (27.7%) patients were classified as adverse-risk. For the ELN 2022 prognosis stratification system, a total of 269 patients were included, with 135 (50.2%) patients classified as favorable-risk, 42 (15.6%) patients as intermediate-risk, and 92 (34.2%) patients as adverse-risk.

**Figure 8:**
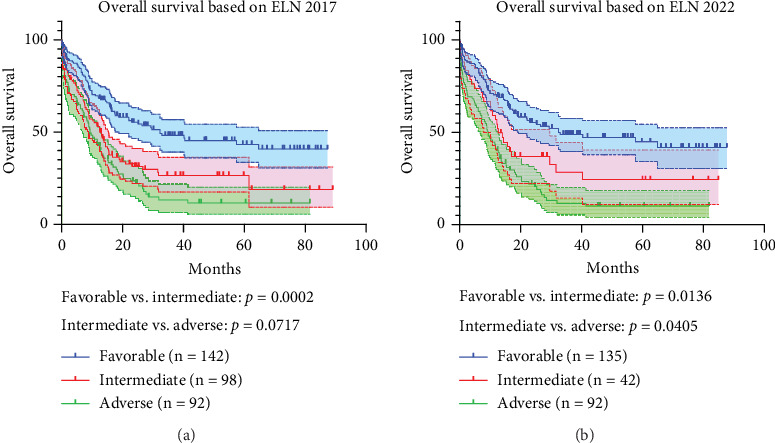
Survival analysis according to ELN 2017 and 2022 guidelines. (a) OS analysis based on ELN 2017. (b) OS analysis based on ELN 2022.

As shown in Figure 8, the 2017 version performs poorly in differentiating between intermediate-risk and adverse-risk patients (*p* = 0.0717, HR: 0.7398, 95% CI: 0.5329–1.027, Figure 8(a)), while the ELN 2022 version not only effectively resolved this issue but also demonstrated excellent performance in classifying favorable-risk and intermediate-risk patients (*p* = 0.0405, HR: 0.6602, 95% CI: 0.4437–0.9823, Figure 8(b)).

#### 3.6. The Impact of Coexistent *KIT* and/or *FLT3* Mutations on the Prognosis of Patients With *RUNX1::RUNX1T1* or *CBFB::MYH11* Fusion Gene

**Figure 9:**
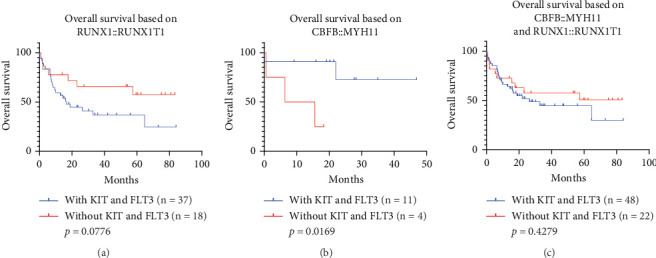
Survival analysis on patients with *RUNX1::RUNX1T1* or *CBFB::MYH11* fusion gene. (a) OS of *RUNX1::RUNX1T1-*positive patients with *KIT* and/or *FLT3* mutations versus without *KIT* and/or FLT3 mutations. (b) OS of *CBFB::MYH11*-positive patients with *KIT* and/or *FLT3* mutations versus without *KIT* and/or *FLT3* mutations. (c) OS of *RUNX1::RUNX1T1*- and *CBFB::MYH11*-positive patients with *KIT* and/or *FLT3* mutations versus without *KIT* and/or *FLT3* mutations.

The ELN 2022 guidelines emphasized that the presence of *KIT* and/or *FLT3* mutations does not alter the favorable prognosis associated with *RUNX1::RUNX1T1* or *CBFB::MYH11*. We have conducted a detailed exploration of this subset of patients. Among the 55 confirmed cases with the *RUNX1::RUNX1T1* fusion gene, patients were divided into two groups based on with or without *KIT* and/or *FLT3* mutations: the concomitant mutation group (*n* = 37) and the nonconcomitant mutation group (*n* = 18). Although no significant difference in prognosis was observed between these two groups (*p* = 0.0776, HR: 1.963, 95% CI: 0.9281–4.152, Figure 9(a)), the trend suggested that patients without *KIT* and/or *FLT3* mutations may have a better prognosis.

Further analysis revealed that among the cases without *KIT* and/or *FLT3* mutations, there were four cases with complex karyotypes, whereas in the presence of these mutations, there were eight cases with complex karyotypes and one with a *TP53* mutation. Fisher's exact test confirmed that there was no significant difference in the distribution of complex karyotypes between these two groups (*p* ≥ 0.05). Therefore, differences in prognosis are primarily influenced by the presence or absence of *KIT* and/or *FLT3* mutations, which is inconsistent with current guidelines.

For patients with *CBFB::MYH11* (*n* = 15), the same analysis was conducted. As shown in Figure 9(b), cases with *KIT* and/or *FLT3* mutations showed a better prognosis (*p* = 0.0169, HR: 0.0606, 95% CI: 0.0061–0.604, Figure 9(a)). Given the small sample size of our data, these findings should be considered preliminary conclusions and interpreted with caution.

Subsequently, when the data from patients positive for either *RUNX1::RUNX1T1* or *CBFB::MYH11* were combined and analyzed together, due to the opposite prognostic trends in the presence of *KIT* and/or *FLT3* mutations, the combined analysis did not show a significant difference in prognosis (*p* = 0.4279, HR: 1.332, 95% CI: 0.6559–2.704, Figure 9(c)).

## 4. Discussion

Here, we present the comprehensive genomic landscape in a Northeast Chinese AML cohort. We first characterized the AML driver variation in the Northeast Chinese population by comparing it with the driver landscape defined in different regional populations and then analyzed the differences between these cohorts. The results showed that the median age of AML occurrence in the United States cohort was 65 years old, which is higher than the median age of 55 years old in our data [27]. The median age for AML at diagnosis in India and Brazil was 42 years old, which was lower than the median age in our population [28, 29]. This may be related to the country's economic status and demographic age structure.

The frequency of *CEBPA* mutation in AML is 6.86%–20.33%, and the frequency of *CEBPA* mutations in Asian people is higher than that in Western countries [13]. In addition, the frequencies of *CEBPA^sm^* and *CEBPA^dm^* in AML patients from the Caucasus population are similar, but *CEBPA^dm^* is more common in Asian populations [13]. Wilhelmson and Porse found that the incidence rate of *CEBPA^dm^* is higher in Asians (6%–15%; average 12%) compared to that in Caucasians (2%–6%; average 4%) [30]. In the population of Southern China, the incidence rates of *CEBPA^dm^* and *CEBPA^sm^* were 14.7% and 3.7%, respectively [31]. In our data, 20.42% of AML patients had *CEBPA* mutations, which was significantly higher than that of Caucasians. For the incidence rate of *CEBPA^dm^*, our data was 11.67%, which was lower than that of the Southern China population. In a Japanese study, *GATA2* was considered a favorable prognostic indicator in *CEBPA^dm^* patients, while *WT1* was associated with a poorer prognosis [32]. However, similar conclusions were not drawn from our data. Our data suggested that patients carrying *GATA2* mutations tend to have a poor prognosis (Figure 5(c)). This may be due to different genetic backgrounds in the impact of *GATA2* and *WT1* mutations on the prognosis of *CEBPA*^dm^ patients, or related to our sample size. Further large-scale studies are warranted to reveal the specific mechanisms and prognostic values of these gene mutations.

To date, the significance of *DNMT3A* mutations in the prognosis of AML is controversial. Three studies from German–Austrian did not reach consistent conclusions [23, 25, 33]. Two of them concluded that *DNMT3A* mutations have no effect on OS, but one study suggested that the comutations of *NPM1*-*DNMT3A*-*NRAS* indicate a better prognosis. In a study conducted in the United States, *DNMT3A* mutation is independently associated with poor prognosis [20]. A study conducted in southern China found that the survival rate of CN-AML patients with *DNMT3A* mutations is lower [24]. In our data, *DNMT3A* independently predicted poor prognosis of AML patients (*p* = 0.0052).

For the specific mutation site, the German–Austrian AML study found that the *DNMT3A* mutation occurred in 20.9% of patients, of whom 64.5% were mutated at R882 [23]. In a US cohort, 22.1% of AML patients had *DNMT3A* mutations, of which 59.7% happened at R882 [20]. In a Japanese cohort, 20.7% of AML patients have *DNMT3A* mutations, of which 53.6% have mutations at R882 [34]. Our data showed that 16.7% of patients were accompanied by *DNMT3A* mutations, which was slightly lower than the above studies. Moreover, 65.1% of them occurred at R882, which was similar to them. In the Japanese cohort, *DNMT3A* R882 is considered an independent predictor of poor prognosis, and similar results were observed in the German cohort [23, 34]. However, our data did not suggest the above conclusion, which is consistent with the conclusions of the United States and France studies [35, 36]. Furthermore, our cohort of AML patients with *DNMT3A* mutations had older age and higher WBC and PLT counts, which was consistent with the study of the German–Austrian cohort [23], but there was no difference in the male-to-female ratio of our cohort, and the BM blast cell percentages were not significantly increased (*p* = 0.0862) [23].

It is well-known that there is a potential association between *DNMT3A* mutation and clonal hematopoiesis of indeterminate potential [37]. In the presence of the *DNMT3A* mutation, these AML driver variations may work together to ultimately lead to the development of AML. Therefore, the impact of *DNMT3A* and other comutations on OS has been a subject of great interest. Further research and understanding of the impact of these comutations on AML prognosis are expected to provide new insights and strategies for precision treatment of AML. A study from the United States indicates that the *DNMT3A* mutation coexisting with high-risk groups of *NPM1*+*FLT3-ITD* mutations leads to poor prognosis [36]. A study from Europe and the United States found that in the presence of *NPM1*mut+*DNMT3A*mut, *FLT3-ITD* mutation is associated with poorer prognosis (*p* = 0.009) [33]. Another study conducted by a Spanish team found that in the presence of *NPM1* mutations, *DNMT3A* mutations may lead to poor clearance of *NPM1* minimal residual disease (MRD). However, *DNMT3A* does not affect the prognosis of patients with *FLT3-ITD*+*NPM1* mutations [38]. Furthermore, our data provided additional confirmation that in terms of OS, the *DNMT3A* mutation had no significant impact on prognosis regardless of whether coexisting with *NPM1* and/or *FLT3-ITD* mutations (Figure 6).

Due to the limited number of newly diagnosed leukemia patients with NGS results (*n* = 713) and with survival information (*n* = 377), our analysis is limited. Regarding the *DNMT3A* mutation, a controversial prognostic mutation for prognostic evaluation, we believe that it was an independent poor prognostic indicator for AML patients in Northeast China. As for the differences in *CEBPA^dm^* in OS analysis between males and females, further analysis with a larger sample size is needed.

In addition to *CEBPA* and *DNMT3A* mutations, we also analyzed the impact of other common gene mutations on OS of AML patients. Most of these mutations showed no independent prognostic effects. Notably, *TP53* and *ASXL1* mutations were also observed to be strongly associated with poor prognosis in our data, consistent with the ELN 2022 guideline [4]. Contrary to several studies in Germany, *RUNX1* mutation did not indicate poor prognosis in our data [39, 40]. Even after excluding favorable prognosis, there are no significant differences, but it can be seen from Figure 7(m) that the *RUNX1* mutation has a trend of poor prognosis (*p* = 0.1269). This difference from the German population may be related to the sample size and different genetic backgrounds, and further research is needed to explore these potential influencing factors. There was no significant difference in the comparison of OS between *NPM1^mut^* and *NPM1^wt^* (*p* = 0.5401), which was consistent with the results of the Japanese population (*p* = 0.6043) [34]. In addition, this Japanese cohort study also showed that patients with *NPM1^mut^* had better prognosis characteristics after excluding abnormal karyotypes (*p* = 0.0634) [34]. However, the *NPM1* mutation group did not show better OS in our cohort (*p* = 0.7972, Figure 7(b)), which was consistent with the results of a South Korean study. In both Southern Chinese and South Korean studies, *NPM1*mut failed to be an independent prognostic factor [41, 42], which may be due to the other factors that were not eliminated. Taken together, the prognostic significance of *NPM1* in East Asian populations requires further studies with a larger sample size.

Compared to the 2017 version, the ELN 2022 guideline incorporated new cytogenetic and molecular events, resulting in a higher number of AML patients being classified as having intermediate-risk or adverse-risk in our study. The ELN 2022 guideline has improved the accuracy of predicting outcomes for intermediate-risk and adverse-risk AML patients [43]. This change aligned with our observations presented in Figure 8. The findings presented in this study aim to provide a reference for prognostic stratification of AML patients in Northeast China. Studies have shown the impact of newly included gene mutations on prognosis, such as *BCOR*, *EZH2*, *SF3B1*, *SRSF2*, *STAG2*, and *U2AF1* in the ELN 2022 guideline [44]. However, due to the incompleteness of our data and the limited sample size, we were unable to conduct a detailed analysis of these genes for the time being, but we will put this as one of our future directions.

Nevertheless, we have some questions regarding the ELN 2022 guideline. Specifically, the guideline mentioned that the presence of *KIT* and/or *FLT3* mutations does not alter the low-risk prognostic outcomes brought by the *RUNX1::RUNX1T1* or *CBFB::MYH11* fusion gene. However, our research data showed a different trend. When the *RUNX1::RUNX1T1* fusion gene is accompanied by other gene mutations, the prognosis of patients showed a worse trend (*p* = 0.0776, Figure 9(a)). For *CBFB::MYH11* fusion genes, the prognosis of patients showed a better trend when accompanied by other gene mutations (*p* = 0.0169, Figure 9(b)). Previous studies have shown that the coexistence of the *CBFB::MYH11* fusion gene with *KIT* and/or *FLT3* mutations is mainly associated with relapse rates rather than OS [45]. Moreover, the presence of *KIT* and/or *FLT3* mutations leads to a poorer prognosis for these two fusion genes [44–46]. Due to the small size of *CBFB::MYH11*-positive cases in our study, our results were inconsistent with literature reports. The more convictive result of the prognostic effects of the *CBFB::MYH11* fusion gene in different mutational backgrounds is to be uncovered in studies with a more expanded sample size. Clinically, there is great hope placed on new advances in gene mutation research and treatment, with some studies providing a stronger theoretical foundation and practical guidance for clinical treatment. For patients with *CEBPA* gene mutation, current studies generally showed that they were highly sensitive to conventional chemotherapy. An important study from Suzhou, China, pointed out that although stem cell transplantation and venetoclax combined with hypomethylating agents (VEN+HMA) represent cutting-edge and vital treatments for a variety of hematological diseases, conventional chemotherapy is still considered the preferred treatment strategy for patients with *CEBPA^bzip^* mutation [47].

On the other hand, *NPM1* gene mutations are common in about 50%–60% of AML patients with abnormal chromosomal karyotype. These patients typically respond well to induction chemotherapy; however, it is important to note that their response to hypomethylating drugs is poor [48]. This result not only deepens our understanding of the biological characteristics of *NPM1*-mutated AML but also provides an important basis for selecting treatment strategies in clinical practice.

For the prognostic significance of *DNMT3A* in this paper, there is almost no literature on the association between commonly used clinical treatment methods and *DNMT3A*, and only sporadic studies on the related mechanism have emerged [49]. *DNMT3A* mutations often occur as accompanying mutations with *NPM1* mutations. However, in our study, *NPM1* mutations appeared to have no significant prognostic significance for East Asian populations. There are few studies on the treatment of AML patients with *DNMT3A* mutations. This suggested that under the genetic background of East Asian populations, related research in this area is worth exploring in depth.

Since Chinese population migration is characterized by a large outflow from the Northeast region and a relatively small inflow from the south, our hospital mainly serves patients from Liaoning Province and a considerable portion of Inner Mongolia. Therefore, we believe that our data reflects gene mutation status in AML patients from Northeast China. We did observe differences in the incidence of *CEBPA* and *DNMT3A* mutations compared with European and American populations, which reminds us that the driver genetic landscape of AML is extremely complex, and it is important to consider ethnic background when stratifying disease risk.

In the population we conducted this study, as a result of different medical insurance coverages and wills to active treatment, not all the patients had continuous and sustained treatment nor costly gene mutation detection; therefore, it is difficult for us to obtain comprehensive data, which led to a limited sample size available in the study. In the near future, we will gather more complete and detailed medical data for these patients, deeply explore the unique information on genetic mutations among populations in Northeast China, and provide more precise and effective strategies and evidence for the treatment of AML.

## Figures and Tables

**Table 1:** The characteristics of 713 AML patients in Northeast China.

	**All patients (** **n** = 713**)**	**Male (** **n** = 362**)**	**Female (** **n** = 351**)**	**p** ** (adj.)**
Age, years, median (IQR)	55 (42–64)	56 (44–64)	55 (41–64)	0.3476
Laboratory examinations, median (IQR)				
BM blast (%)	58.3 (38.4–75.6)	59.2 (38.4–76)	57.2 (37–74.4)	0.6433
WBC (×10^9^/L)	14.6 (3.8–48.8)	17.12 (4.22–55.13)	12.4 (3.49–44.55)	0.3567
PLT (×10^9^/L)	40 (18–79.75)	40 (18–79)	40 (18.25–80.75)	0.7422
HGB (×G/L)	77 (63.25–97)	80 (64.25–103.8)	74.5 (62–91)	0.0004
Cytogenetics, *n* (%)				
Normal karyotype	362 (50.8%)	170 (47.0%)	192 (54.7%)	0.047
Abnormal karyotypes	270 (37.9%)	153 (42.3%)	117 (33.3%)	
Unknown	81 (11.4%)	39 (10.8%)	42 (11.2%)	
FAB subtypes, *n* (%)				
M0	3 (0.4%)	3 (0.8%)	0 (0.0%)	0.706
M1	43 (6.0%)	20 (5.5%)	23 (6.6%)	
M2	277 (38.8%)	140 (38.7%)	137 (39.0%)	
M4	153 (21.5%)	76 (21.0%)	77 (21.9%)	
M5	230 (32.3%)	118 (32.6%)	112 (31.9%)	
M6	2 (0.3%)	1 (0.3%)	1 (0.3%)	
M7	1 (0.1%)	1 (0.3%)	0 (0.0%)	
Unknown	4 (0.6%)	3 (0.8%)	1 (0.3%)	
Gene mutations, *n* (%)				
*NPM1*	155 (21.7%)	70 (19.3%)	85 (24.2%)	0.123
*FLT3-ITD*	145 (20.3%)	70 (19.3%)	75 (21.4%)	0.516
*FLT3-TKD*	56 (7.9%)	32 (8.8%)	24 (6.8%)	0.333
*ASXL1*	82 (11.5%)	51 (14.1%)	31 (8.8%)	0.034
*TET2*	117 (16.4%)	54 (14.9%)	63 (17.9%)	0.312
*KIT*	80 (11.2%)	41 (11.3%)	39 (11.1%)	1.000
*DNMT3A*	117 (16.4%)	58 (16.0%)	59 (16.8%)	0.840
*IDH1*	51 (7.2%)	18 (5.0%)	33 (9.4%)	0.028
*IDH2*	77 (10.8%)	40 (11.0%)	37 (10.5%)	0.904
*EZH2*	16 (2.2%)	10 (2.8%)	6 (1.7%)	0.450
*RUNX1*	52 (7.3%)	33 (9.1%)	19 (5.4%)	0.062
*TP53*	41 (5.8%)	23 (6.4%)	18 (5.1%)	0.523
*NARS*	121 (17.0%)	72 (19.9%)	49 (14.0%)	0.022
*CEBPA*	131 (18.4%)	64 (17.7%)	67 (19.1%)	0.630

**Table 2:** Clinical characteristics of male and female patients with *CEBPA^dm^.*

	**Male (** **n** = 18**)**	**Female (** **n** = 21**)**	**p** ** (adj.)**
Age, year, median (IQR)	46 (27.8–53.5)	55 (36–62)	0.2310
Laboratory examinations, median (IQR)			
BM blast (%)	57.6 (41.00–68.60)	46.4 (37.3–64.20)	0.3773
WBC (×10^9^/L)	9.85 (8.80–29.55)	11.90 (4.61–40.4)	0.3690
PLT (×10^9^/L)	29.50 (12.50–47.25)	20 (9–37)	0.4384
HGB (×g/L)	104.5 (76.75–112.80)	82 (70.0–102.0)	0.1406
FAB subtypes, *n* (%)			
M1	1 (5.6%)	5 (23.8%)	0.368
M2	13 (72.2%)	12 (57.1%)	
M4	4 (22.2%)	4 (19.0%)	
Cytogenetics, *n* (%)			
Normal karyotype	16 (88.9%)	15 (71.4%)	0.501
Abnormal karyotype	1 (5.6%)	4 (19.0%)	
Unknown	1 (5.6%)	2 (9.5%)	
Fusion gene positive	0	0	

**Table 3:** Clinical characteristics of patients with *DNMT3A* mutations.

	***DNMT3A*** ^***wt***^ ** (** **n** = 314**)**	***DNMT3A*** ^***mut***^ ** (** **n** = 63**)**	**p** ** (adj.)**
Age, year, median (IQR)	53 (36.62)	59 (53, 63)	< 0.001
Male (%)	144 (45.9%)	31 (49.2%)	0.679
Female (%)	170 (54.1%)	32 (50.8%)	
Laboratory examinations, median (IQR)			
BM blast (%)	61.8 (39.1, 76.5)	54.4 (38, 66.4)	0.0862
WBC (×10^9^/L)	11.25 (3.3, 43.87)	33.9 (9, 64.6)	0.0015
PLT (×10^9^/L)	38 (17, 70)	58 (24, 97)	0.0048
HGB (×g/L)	78 (64, 101)	77 (66, 94)	0.9001
Cytogenetics, *n* (%)			
Normal karyotype	159 (50.6%)	39 (61.9%)	0.118
Abnormal karyotypes	127 (40.4%)	17 (27.0%)	
Unknown	28 (8.9%)	7 (11.1%)	
Comutations			
*FLT3-ITD*, *n* (%)	56 (17.8%)	23 (36.5%)	0.002
*NPM1*, *n* (%)	53 (16.9%)	32 (50.8%)	< 0.001
FAB subtypes, *n* (%)			
M1, *n* (%)	24 (7.6%)	1 (1.6%)	< 0.001
M2, *n* (%)	141 (44.9%)	13 (20.6%)	
M4, *n* (%)	71 (22.6%)	12 (19.0%)	
M5, *n* (%)	76 (24.2%)	37 (58.7%)	
M7, *n* (%)	1 (0.3%)	0 (0.0%)	
AML, *n* (%)	1 (0.3%)	0 (0.0%)	

**Table 4:** Univariate Cox regression analysis of factors influencing patient's survival.

	**B**	**SE**	**Wald**	**df**	**Sig.**	**Exp(** **B** **)**	**95% confidence interval**	
							**Lower**	**Upper**
Age	0.034	0.005	53.955	1	< 0.001	1.035	1.026	1.044
Gender	0.162	0.126	1.637	1	0.201	1.175	0.918	1.506
WBC (×10^9^/L)	0.002	0.001	6.18	1	0.013	1.002	1.001	1.004
PLT (×10^9^/L)	0	0.001	0.049	1	0.825	1	0.998	1.002
HBG (×g/L)	−0.006	0.003	5.394	1	0.02	0.994	0.989	0.999
PB blast (%)	0.003	0.002	2.198	1	0.138	1.003	0.999	1.007
BM blast (%)	0.006	0.003	4.452	1	0.035	1.006	1	1.012

**Table 5:** Multivariate Cox regression analysis identifying independent predictors of patient's survival.

	**B**	**SE**	**Wald**	**df**	**Sig.**	**Exp(** **B** **)**	**95% confidence interval**	
							**Lower**	**Upper**
Age	0.036	0.005	52.408	1	< 0.001	1.037	1.027	1.047
PB blast (%)	0.004	0.003	1.969	1	0.161	1.004	0.998	1.009
BM blast (%)	0.008	0.004	5.160	1	0.023	1.009	1.001	1.016
HGB (×g/L)	−0.009	0.003	9.096	1	0.003	0.991	0.986	0.997
WBC (×10^9^/L)	0.002	0.001	2.471	1	0.116	1.002	1.000	1.004
PLT (×10^9^/L)	0.001	0.001	1.070	1	0.301	1.001	0.999	1.003
*NPM1*	−0.463	0.169	7.489	1	0.006	0.629	0.452	0.877
*FLT3-ITD*	−0.147	0.170	0.745	1	0.388	0.864	0.619	1.205
*ASXL1*	0.156	0.199	0.614	1	0.433	1.169	0.791	1.726
*DNMT3A*	0.316	0.168	3.551	1	0.059	1.371	0.987	1.904
*CEBPA^bZIP-inf^*	−0.630	0.233	7.290	1	0.007	0.533	0.337	0.841
*TP53*	1.309	0.291	20.224	1	< 0.001	3.701	2.092	6.547
*EZH2*	−0.477	0.725	0.432	1	0.511	0.621	0.150	2.572

## Data Availability

The clinical data of patients used to support the findings of this study are available from the corresponding author upon request.

## Supporting Information

Supporting Information Additional supporting information can be found online in the Supporting Information section. Figure S1: OS of the patients younger than 30 years old versus the patients between 30 and 39 years old. For Table 1, descriptive analysis was conducted on clinical data such as age, WBC count, and PLT count for all patients, male patients, and female patients. Additionally, t-tests were performed to compare these indicators between male and female patients. Chi-square tests were used to compare chromosomal changes, FAB classifications, and mutation status between male and female patients. For Figures 1, 3, 4, 5, 6, 7, 8, and 9 and Table 4, univariate Cox proportional hazards regression analysis was conducted.  Table 5 presents the results of multivariate Cox proportional hazards regression analysis. Chi-square tests were utilized for Tables 2 and 3.
