# Supplementary material for: Gene Mutation Characteristics and Prognostic Significance in Acute Myeloid Leukemia Patients From Northeast China
Source: Hum Mutat. 2025 Feb 20;2025:7730186. doi: 10.1155/humu/7730186 (PMC11918257; doi:10.1155/humu/7730186)
Supplement: Supporting Information — Additional supporting information can be found online in the Supporting Information section. Figure S1: OS of the patients younger than 30 years old versus the patients between 30 and 39 years old. For Table 1, descriptive analysis was conducted on clinical data such as age, WBC count, and PLT count for all patients, male patients, and female patients. Additionally, t-tests were performed to compare these indicators between male and female patients. Chi-square tests were used to compare chromosomal changes, FAB classifications, and mutation status between male and female patients. For Figures 1, 3, 4, 5, 6, 7, 8, and 9 and Table 4, univariate Cox proportional hazards regression analysis was conducted. Table 5 presents the results of multivariate Cox proportional hazards regression analysis. Chi-square tests were utilized for Tables 2 and 3. [file 7730186.f1.docx]

For Table 1, descriptive analysis was conducted on clinical data such as age, white blood cell count, and platelet count for all patients, male patients, and female patients. Additionally, t-tests were performed to compare these indicators between male and female patients. Chi-square tests were used to compare chromosomal changes, FAB classifications, and mutation status between male and female patients.

For Figures 1, 3, 4, 5, 6, 7, 8, 9, and Table 4, univariate Cox proportional hazards regression analysis was conducted. Table 5 presents the results of multivariate Cox proportional hazards regression analysis.

Chi-square tests were utilized for Table 2 and Table 3.


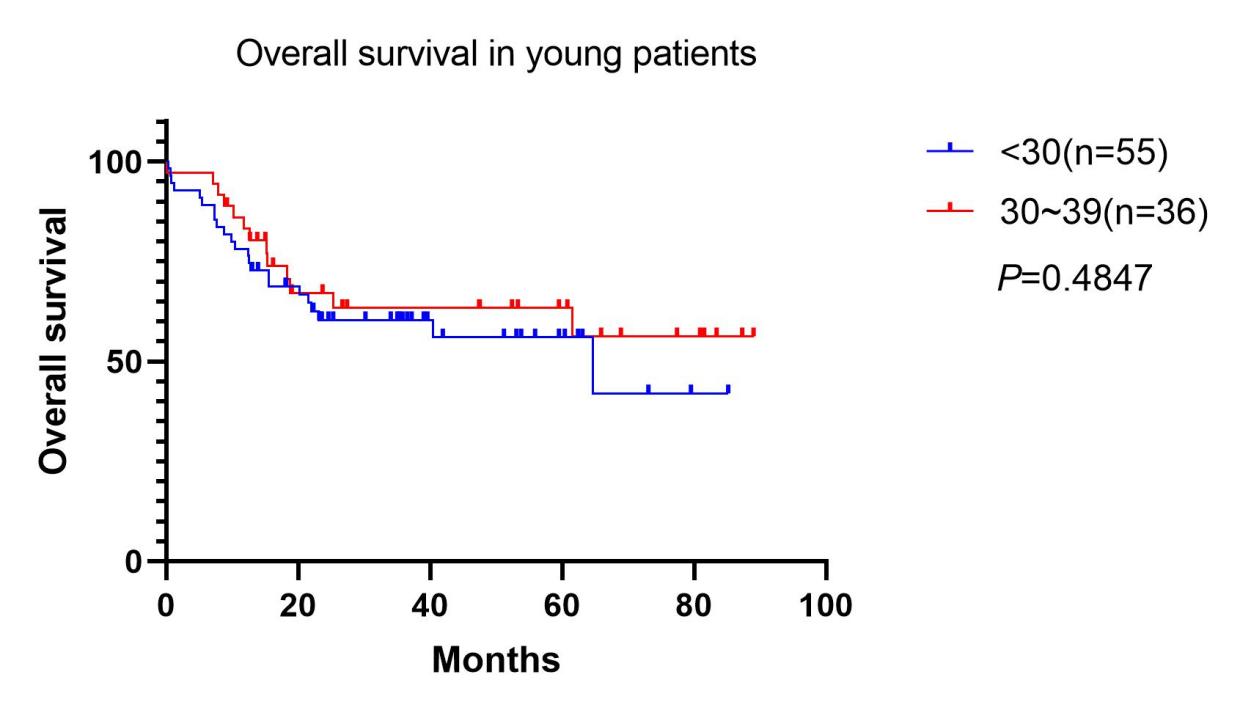


figure 1 OS of the patients younger than 30 years old vs。the patients between 30 to 39 years old
